# Prostate-Specific Membrane Antigen PET-Guided Intensification of Salvage Radiotherapy After Radical Prostatectomy: A Phase 2 Randomized Clinical Trial

**DOI:** 10.1001/jamaoncol.2025.3746

**Published:** 2025-10-02

**Authors:** Colin Belliveau, Fred Saad, Danny Duplan, Claire Petit, Guila Delouya, Daniel Taussky, Maroie Barkati, Carole Lambert, Marie-Claude Beauchemin, Sebastien Clavel, Gary Mok, Levon Igidbashian, Anne-Sophie Gauthier-Paré, Thu-van Nguyen, Pierre-Yves McLaughlin, Khun Visith Keu, Jean N. DaSilva, Daniel Juneau, Cynthia Ménard

**Affiliations:** 1 Radiation Oncology, Centre Hospitalier de l’Université de Montréal (CHUM), Montréal, Québec, Canada; 2 Urology, Centre Hospitalier de l’Université de Montréal (CHUM), Montréal, Quebec, Canada; 3 Département de radio-oncologie, Centre Intégré de Santé et de Services Sociaux de Laval, Laval, Québec, Canada; 4 Radiotherapy, Institut Paoli Calmettes, Marseille, France; 5 Radiation Oncology, Hôpital de Charles-Le Moyne, Longueuil, Québec, Canada; 6 Radiation Oncology, Hôpital de Gatineau, Gatineau, Québec, Canada & University of Ottawa, Division of Radiation Oncology; 7 Nuclear Medicine, Hôpital de la Cité-de-la-Santé, Laval, Québec, Canada; 8 Radiochemistry and Cyclotron Laboratory, Centre de Recherche du Centre Hospitalier de l’Université de Montréal (CRCHUM); 9 Nuclear Medicine, Centre Hospitalier de l’Université de Montréal (CHUM), Montréal, Québec, Canada; 10 Radiation Oncology, Centre de Recherche du Centre Hospitalier de l’Université de Montréal (CRCHUM), Montréal, Québec, Canada

## Abstract

**Importance:**

Prostate-specific membrane antigen positron emission tomography (PSMA-PET) offers superior accuracy in detecting prostate cancer lesions leading to intensified radiotherapy (RT), but its impact on patient outcomes is still undefined.

**Objective:**

To evaluate whether intensification of salvage RT (SRT) after radical prostatectomy (RP) guided by PSMA-PET (PSMAiSRT) is associated with improved failure-free survival (FFS).

**Design, Setting, and Participants:**

PSMAiSRT was a stratified cohort within a larger PSMA-guided intensification of radiotherapy (PSMAgRT) trial, a phase 2, two-center, registry-based randomized clinical trial. Patients with biochemical recurrence following RP who were eligible for standard-of-care (SOC) SRT from May 2018 to February 2021, were eligible for randomization in the PSMAiSRT stratum. A total of 130 patients were randomized, with 2 who did not proceed to radiotherapy (RT). The cutoff date for the primary analysis was October 26, 2023

**Intervention:**

Patients were randomized in a 1:1 ratio to receive either SOC SRT to the prostate bed, with or without elective pelvic RT, with or without adjuvant hormonal therapy (HT), or PSMA-PET/CT-guided SRT, intensified to detected sites of disease.

**Main outcome and measures:**

The primary end point was FFS, defined as PSA progression (PSA nadir >0.2 ng/mL), radiological progression, next-line therapy initiation, or death.

**Results:**

Among 128 patients (median [IQR] age, 71 [64-74] years), median (range) PSA at enrollment was 0.3 (0.1-3.0) ng/mL. In the PSMAiSRT group, 33 of 64 patients (52%) received intensified SRT; with addition of pelvic RT (n = 16 [25%]), metastasis-directed RT (n = 2 [3%]), lymph node boost (n = 19 [30%]), or prostate bed boost (n = 15). Adjuvant hormone therapy was equally prevalent in both arms (55 [86%] control vs 54 [84%] PSMAiSRT). At a median (range) follow-up of 37 (7-60) months, PSMAiSRT improved FFS (hazard ratio [HR], 0.50; 95% CI, 0.27-0.94; *P* = .04) and eugonadal FFS (HR, 0.45; 95% CI, 0.21-0.96; *P* = .03), with its greatest benefit in the subgroup with PSA of 0.3 ng/mL or more (HR, 0.17; 95% CI, 0.04-0.79; *P* = .01). Fewer next-line treatment events occurred in the PSMAiSRT arm (4 vs 12; HR, 0.32; 95% CI, 0.11-1.02; *P* = .04). There were no significant differences in toxic effects or quality of life between arms.

**Conclusion and Relevance:**

This phase 2 trial demonstrated an isotoxic improvement in cancer control with PSMA-PET-guided intensification of SRT after RP. Confirmatory evidence is awaited from a subsequently accrued phase 3 trial.

**Trial Registration:**

ClinicalTrials.gov NCT03525288

## Key Points

**Question:**

Does prostate-specific membrane antigen positron emission tomography (PSMA-PET)–guided intensification of salvage radiotherapy (SRT) after radical prostatectomy (RP) improve failure-free survival (FFS) in patients with biochemical recurrence?

**Findings:**

In this phase 2 randomized clinical trial, including 64 patients in the treatment arm and 64 in the control group, PSMA-PET–guided intensification of SRT was associated with significantly improved FFS compared with standard-of-care SRT. The treatment was isotoxic, with comparable quality of life between groups.

**Meaning:**

PSMA-PET may guide intensification of salvage radiotherapy after prostatectomy.

## Teaser

This randomized clinical trial examines the association of prostate-specific membrane antigen positron emission tomography (PET)–guided intensification of salvage radiotherapy with failure-free survival among patients with prostate cancer after radical prostatectomy.

## Introduction

After radical prostatectomy (RP), some patients will experience a biochemical recurrence (BCR).^1^ Recent studies have reported an advantage in both metastasis-free and biochemical relapse–free survival in adopting early salvage radiation therapy (SRT) on detecting BCR following RP.^2^ Even though SRT at BCR after RP has shown excellent oncologic outcomes,^3,4^ more than a quarter of patients can experience subsequent BCR.^5^

Prostate-specific membrane antigen positron emission tomography (PSMA-PET) has been shown to be superior to conventional imaging after RP^6^ with excellent diagnostic accuracy in patients presenting with BCR after RP.^7,8^ Prior studies suggest that PSMA-targeted ligand PET can considerably alter the administration of SRT, with modifications in up to 50% of cases.^9,10,11^ However, the impact of such a change on outcomes is unknown.

Given the high sensitivity and specificity of PSMA-PET, the hypothesis of this study was that PSMA-PET–guided intensification of salvage radiation therapy (PSMAiSRT) would improve failure-free survival (FFS) compared with conventional SRT in patients presenting with BCR after RP.

## Methods

### Study Design and Participants

The trial protocol is available in Supplement 1. PSMAiSRT was a stratified cohort within the larger PSMA-guided intensification of radiotherapy (PSMAgRT)^12^ trial, a phase 2, two-center, cohort multiple randomized clinical trial (1:1 randomization ratio, NCT03525288), which was embedded in a prospective registry (NCT03378856). In the PSMAgRT trial, patients planned for definitive radiotherapy (RT) who were at risk of undetected regional or distant metastases on conventional imaging were enrolled. This included patients with oligometastatic disease on conventional imaging, newly diagnosed patients with localized high-risk disease, those undergoing salvage post-RT, and PSMAiSRT. Between May 2018 and February 2021, these patients were randomized within their specific strata.

**Figure 1.**
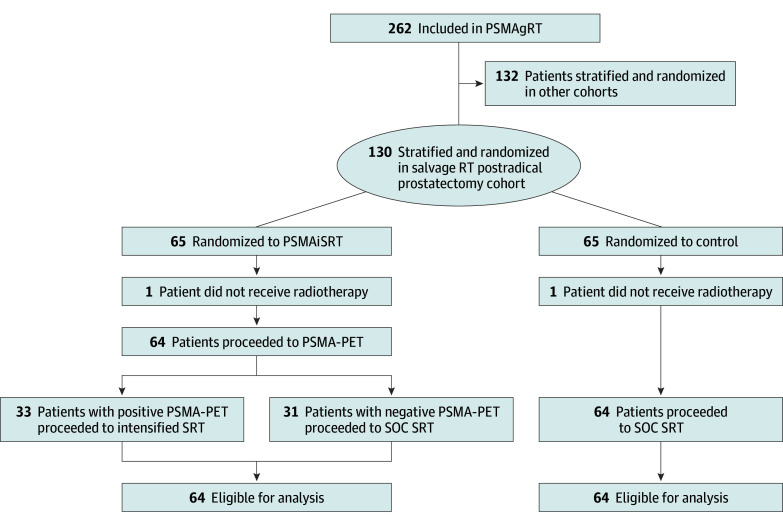
Consort Diagram Showing Enrollment, Randomization, and Completion of Treatment PSMAgRT indicates PSMA-guided radiotherapy; PSMAiSRT, PSMA, intensification of salvage radiotherapy; PSMA-PET, prostate-specific membrane antigen positron emission tomography; RT, radiotherapy; SOC, standard of care; SRT, salvage radiotherapy.

We report the early findings of the PSMAiSRT strata meeting its primary end point in a triggered interim analysis. Those in the experimental arm underwent PSMA-PET with intensified RT based on PSMA-PET findings. Patients were eligible if planned for salvage RT with biochemical recurrence greater than 0.1 ng/mL, an Eastern Cooperative Oncology Group (ECOG) score of 0 to 1, and a Charlson Comorbidity Index score lower than 5. Exclusions included prior androgen deprivation therapy within 12 months and prior PSMA-PET. Written consent was obtained for every patient. The CONSORT diagram for enrollment of the PSMAiSRT strata is shown in Figure 1. This study followed the Consolidated Standards of Reporting Trials (CONSORT) reporting guidelines.^13^ The protocol was approved by the institutional review board at each participating institution, and all patients provided written informed consent.

### Randomization

Variable block randomization (block size 4, 6, 8) was done as a cohort multiple randomized controlled trial and executed by an eClinical Data Management platform (CastorEDC) after receiving approval from the ethics committee. Patients initially consented to participate in the registry (NCT03378856), and only those meeting eligibility and randomized to the experimental group provided additional consent for PSMAiSRT. Analysis was according to intent-to-treat study arm assignment, and no treatment crossovers occurred in the trial.

### Procedures

Physicians recorded their treatment intention electronically before randomization, including their RT target volumes (prostate bed with or without elective pelvic lymph nodes) as well as adjuvant androgen deprivation use and duration. Standard-of-care SRT included the prostate bed and, at the discretion of the radiation oncologist, could also encompass the pelvic lymph nodes, based on computed tomographic (CT) findings alone and without PSMA-PET. If hormonal therapy (HT) was administered, it was initiated after PSMA-PET and prior to the start of RT. As a pragmatic trial design, no strict criteria were established for selecting RT volumes or for determining which patients would receive hormonal therapy, allowing treatment to reflect care as usual.

In the experimental arm (PSMAiSRT), RT was intensified (volume and/or dose) based on PSMA-PET results. The equivalent dose in 2-Gy fractions dose (α/β 1.4) for newly detected sites was up to 66 Gy for lymph nodes or oligo-metastases and up to 77 Gy for prostate bed recurrences, while respecting organ-at-risk limits. Patients with PSMA-PET–avid lymph nodes received whole pelvic elective RT with an added boost to the avid nodes. PSMAiSRT patients without suspicious lesions on PSMA-PET results underwent standard-of-care SRT. HT was administered per standard of care, and systemic therapy management was not altered unless widely metastatic disease was detected.

All patients completed quality of life assessments (Expanded Prostate Cancer Index Composite for Clinical Practice [EPIC-CP], Functional Assessment of Cancer Therapy–Prostate [FACT-P], and International Prostate Symptom Score [IPSS]) at baseline and annually. Acute and late-grade 2 or higher toxic events were monitored using the National Cancer Institute Common Terminology Criteria for Adverse Events (NCI CTCAE) version 5.0 at each clinic visit. PSA assessments were performed per standard of care after SRT (at least once annually). CT abdomen/pelvis, technetium-99m bone scans, and PSMA-PET were conducted for restaging subsequent biochemical failure at the physician’s discretion. The next line of therapy was initiated based on the treating physician’s judgment.

Acquisition details of 18F-DCFPyL PSMA-PET studies have been reported and published previously.^12,14^ Board-certified nuclear medicine physicians interpreted PSMA-PET/CT images identifying probable and definite lesions for RT intensification.

### Outcomes

The primary end point was FFS, defined as the first occurrence of biochemical progression (PSA nadir >0.2 ng/mL; to convert to µg/L, multiply by 1), clinical progression, initiation of next-line therapy, or death. Secondary end points included metastasis-free survival assessed by conventional imaging (bone scan, CT). PSMA-PET was offered to patients at BCR; however, it was not incorporated into the MFS analysis. Secondary end points also included overall survival, biochemical progression-free survival (BPFS), next-line treatment-free survival (TFS), adverse events, and quality of life (QoL).

### Statistical Analysis

The primary FFS end point was measured from the last day of RT to the first event. Estimated FFS at 5 years for RP strata was 40%.^15^ Considering the enhanced diagnostic yield of PSMA-PET, with a 15% increase in sensitivity for bone lesions and a 50% increase for lymph node metastases,^16^ the study, designed with an α of 0.10, 80% power, a hazard ratio of 0.7, and a 1-sided log-rank test, required the enrollment of 117 patients in the PSMAiSRT strata (and 260 in the overarching PSMAgRT trial), accounting for potential ineligibility and follow-up losses. Power analysis was based on stratified analysis.

Kaplan-Meier curves were compared with the log-rank test. Cox regression models estimated the hazard ratio and its 95% CI. Median follow-up was calculated from the end of RT to the last physician assessment or PSA measurement. All *P* values were 2 sided.

TFS was defined as the time from RT completion to subsequent treatment initiation (hormonal therapy with/without ARPI and/or additional RT) and BPFS was defined as the time from treatment completion to the first PSA rise of 0.2 ng/mL or more, aligning with a national phase 3 randomized clinical trial^17^ and PSMA-PET’s high detection rate at this threshold.^18^ Exploratory analysis included eugonadal FFS, measured from achieving testosterone levels of 5.2 ng/L or higher (to convert to µg/L, multiply by 1) until disease progression. If failure occurred before eugonadal levels, the event was counted at time 0. Patients without available testosterone levels and no failure events were censored at time 0 and excluded from the analysis of median time to failure after eugonadal testosterone.

Post hoc analyses assessed freedom from progression based on median pretreatment PSA and stratification factors (grade group and pathologic risk). Toxic effects analysis assessed the cumulative incidence of grade 2 or higher and grade 3 or higher CTCAE v5.0 events at 6 months (acute) and median follow-up (late). Acute toxic effects were assessed within the first 6 months to ensure all patients had at least 1 follow-up visit after completing RT. Event-free survival generated with Kaplan-Meier was calculated from the start of RT to the first toxic event, with separate analyses for overall, gastrointestinal, and genitourinary toxic effects. Toxic events were compared using χ^2^ and Fisher exact tests.

For QoL analysis, minimally important differences (MIDs) in EPIC change scores of 2 points per domain (urinary, bowel, sexual, hormonal) were used as events.^19^ A χ^2^ test was conducted to compare the groups. Data analysis was performed using SPSS statistical software (version 29.0, IBM Corp) for bar plots and R statistical software (version 4.3.1, R Foundation for Statistical Computing) for survival curves and χ^2^ analysis, with the cut off date for analysis set to October 26, 2023.

## Results

### Patients

Among 128 patients the median (IQR) age was 71 (64-74) years. Standard-of-care dose and fractionation varied due to the COVID-19 pandemic and are described in Table 1. Between May 2018 and February 2021, 130 male patients were enrolled and randomized across 2 institutions (Table 2 reports population characteristics). Two patients were excluded because they did not proceed to RT, leaving 128 eligible for analysis. Of these, 64 were assigned to the experimental arm. Median (range) PSA was 0.3 (0.1-3) ng/mL. Intensified SRT was delivered to 33 of 64 imaged participants (52%). Before randomization, 19 (30%) experimental patients were planned for pelvic RT, but with PSMA-PET guidance, 35 (55%) received pelvic SRT, compared with 31 (48%) in the control arm (ns). In addition, 2 patients (3%) received metastasis-directed RT to a bone lesion, 19 (30%) a pelvic lymph node boost, and 15 (23%) a prostate bed boost. Adjuvant HT was used in 55 (86%) of the control arm and 54 (84%) of the PSMAiSRT arm (ns). All patients received prostate bed RT, with or without regional or metastatic-directed treatment because none were found to have widely metastatic disease on PSMA-PET results. The fractionation schedules used are described in Table 1. One patient in the PSMAiSRT group had adjuvant HT added after randomization, with no changes in the control group. Detailed description of PSMA-PET results and their effect on RT are published elsewhere.^12^ eTable 1 in Supplement 2 presents the eugonadal status of patients who received HT. The duration of HT is detailed in eTable 2 in Supplement 2.

**Table 1.** Radiotherapy Dose, Fractionation, and Intensification of Radiotherapy

Variable	No. (%)	
	PSMAiSRT	Control
**RT dose and fractionation**		
Prostate bed	64 (100)	64 (100)
66.0 Gy in 33 fractions	60 (94)	62 (97)
60.0 Gy in 30 fractions	1 (2)	1 (2)
52.4 Gy in 20 fractions	3 (5)	1 (2)
Elective pelvic lymph node	35 (35)	31 (48)
48 Gy in 24 fractions	2 (6)	0
46 Gy in 23 fractions	4 (11)	4 (13)
45 Gy in 25 fractions	1 (3)	0
44 Gy in 22 fractions	27 (77)	27 (87)
42 Gy in 20 fractions	1 (3)	0
**RT intensification based on PSMA-PET**		
No.	64 (100)	64 (100)
Boost to lymph node		
Aim of EQD2 (α/β 1.4) 66 Gy	19 (30)	0
Boost to prostate bed		
Aim of EQD2 (α/β 1.4) 77 Gy	15 (23)	0
Metastasis-directed RT		
Aim of EQD2 (α/β 1.4) 66 Gy	2 (3)	0

Abbreviations: EQD2, equivalent dose in 2-Gy fractions; PSMAiSRT, prostate-specific membrane antigen positron emission tomography guided by PSMA-PET, prostate-specific membrane antigen positron emission tomography; RT, radiotherapy.

**Table 2.** Baseline Characteristics of the Population

Characteristic	No. (%)	
	PSMAiSRT	Control
Grade group		
1	3 (4.7)	1 (1.6)
2	23 (35.9)	28 (43.8)
3	21 (32.8)	27 (42.2)
4	3 (4.7)	5 (7.8)
5	14 (21.9)	3 (4.7)
(p)T stage		
T1c	1 (1.6)	0
T2a	7 (10.9)	8 (12.5)
T2b	4 (6.3)	4 (6.3)
T2c	12 (18.8)	12 (18.8)
T3a	22 (34.4)	24 (37.5)
T3b	17 (26.6)	16 (25.0)
T4	1 (1.6)	0
(p)N stage		
N0	26 (40.6)	32 (50.0)
N1	7 (10.9)	5 (7.8)
Nx	31 (48.4)	27 (42.2)
Age, median (range), y	71 (51-86)	71 (50-82)
PSA, ng/mL		
< Median 0.3	36 (56.0)	37 (58.0)
≥ Median 0.3	28 (44.0)	27 (42.0)
PSA, median (range), ng/mL	0.3 (0.1-2.4)	0.3 (0.1-3.0)
Positive margins	34 (53.1)	20 (31.5)
Time since previous RT/surgery, median (range), mo	19.5 (1.0-188.0)	19.9 (1.4-130.8)

Abbreviations: PSA, prostate-specific antigen (to convert to µg/L, multiply by 1); PSMAiSRT, prostate-specific membrane antigen positron emission tomography guided by prostate-specific membrane antigen positron emission tomography; RT, radiotherapy.

### Primary End Point

At a median (range) follow-up of 37 (7-60) months, PSMAiSRT improved FFS (control, 20 vs PSMAiSRT, 10 events; *P* = .04; hazard ratio [HR], 0.46; 95% CI, 0.22-0.99). Overal, 27 events (90%) were BCR, with 1 initiation of next-line therapy due to rapid PSA doubling (3%) and 2 unrelated deaths (6.7%). No differences were seen in distant metastasis (control, 4 vs PSMAiSRT, 5) or overall survival (1 death per arm).

### Secondary Efficacy End Points and Exploratory Subgroup Analyses

In secondary analysis, differences between groups did not meet conventional levels of statistical significance for biochemical relapse-free survival (control, 18 vs PSMAiSRT, 9 events; *P* = .06; HR, 0.47; 95% CI, 0.21-1.05). TFS favored PSMAiSRT (control, 12 vs PSMAiSRT, 4 events; HR, 0.32; 95% CI, 0.10-1.00; *P* = .04). Next-line therapy included metastasis-directed RT with HT (5 [31%]), HT monotherapy (8 [50%]), or HT with ARPI (3 [19%]). Among the patients who experienced recurrence after PSMAiSRT, half were PSMA-PET positive and benefited from subsequent RT.

**Figure 2.**
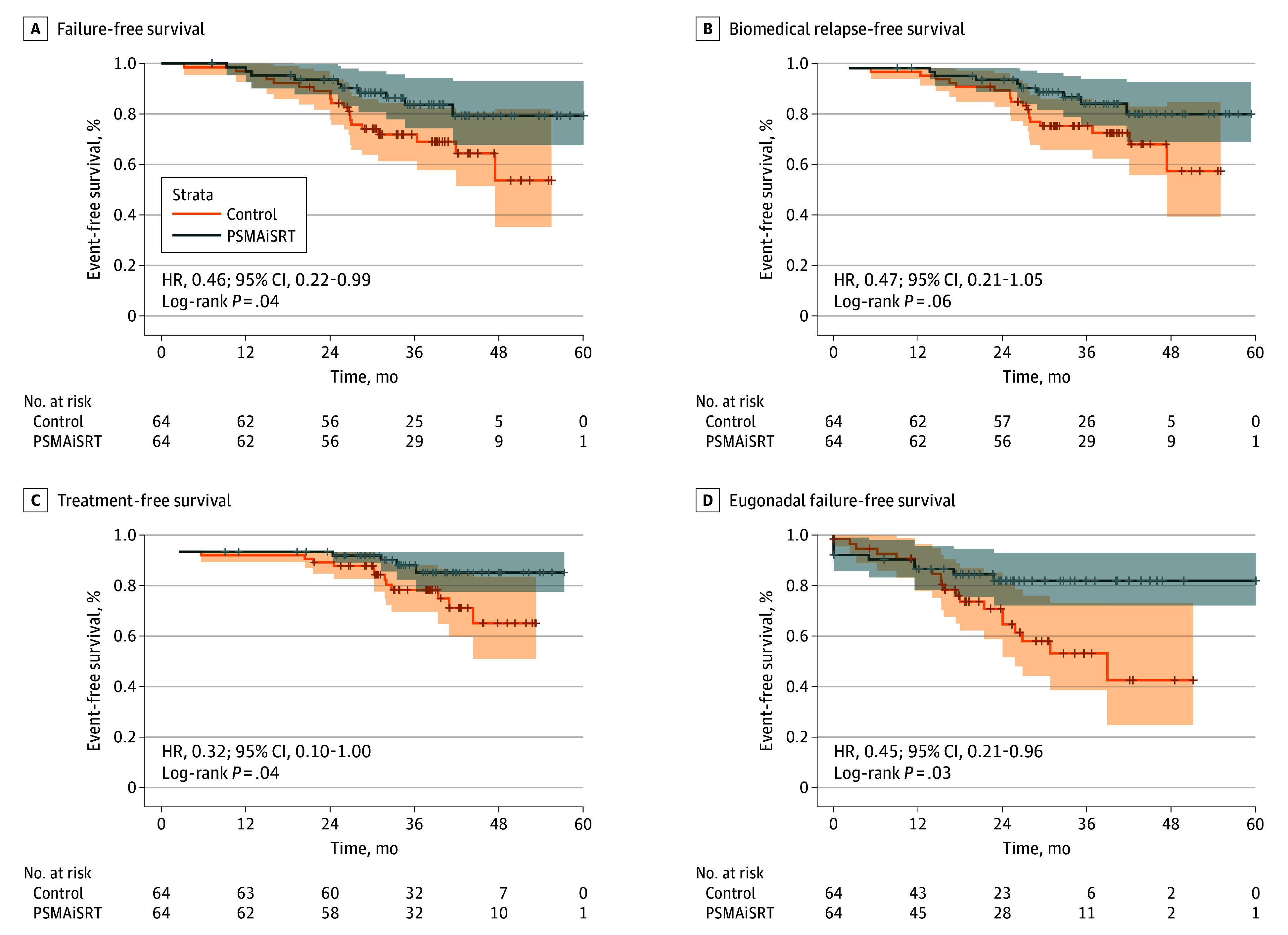
Primary and Key Secondary End Points HR indicates hazard ratio; PSMAiSRT, prostate-specific membrane antigen positron emission tomography guided by prostate-specific membrane antigen positron emission tomography.

Recovery to eugonadal testosterone levels (≥5.2 ng/dL) occurred in 86 men (67%). The median (range) time from achieving eugonadal testosterone levels to failure was 22.3 (0-90) months. Eugonadal FFS was significantly longer for PSMAiSRT (HR, 0.45; 95% CI, 0.21-0.96; *P* = .03). KM curves for FFS, biochemical relapse-free survival, TFS and eugonadal FFS are in Figure 2.

eTable 3 in Supplement 2 compares sites of subsequent failure on PSMA-PET. In the PSMAiSRT group (n = 10), 5 (50%) had distant failures, 1 (10%) out-of-field lymph node failures, and 4 (40%) no detected lesions. In the control group (n = 20), 5 (25%) had out-of-field locoregional failures, 7 (35%) distant failures, and 8 (40%) no lesions.

**Figure 3.**
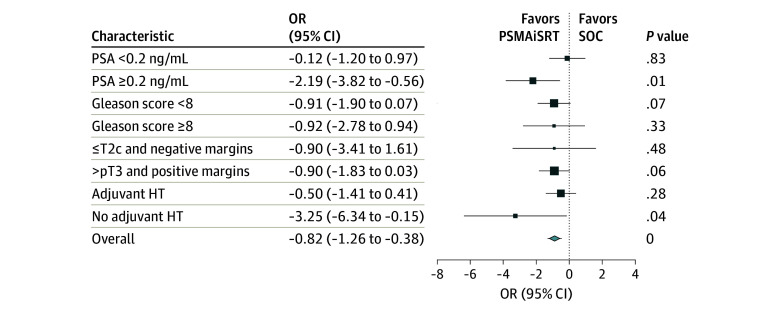
Forest Plot Showing Which Patients Most Benefit From PSMAiSRT Forest plot showing the association of treatment with failure-free survival. PSMAiSRT indicates prostate-specific membrane antigen positron emission tomography guided by prostate-specific membrane antigen positron emission tomography. For prostate-specific antigen, to convert ng/mL to µg/L, multiply by 1.

In subset analysis, the forest plot in Figure 3 and Kaplan-Meier curves (eFigure 1 in Supplement 2), show that when PSA was less than or equal to the median, there was not a significant difference between arms. However, when the median PSA level was 0.3 ng/mL or higher, FFS for PSMAiSRT was significantly greater than the control arm (n = 55 [43%]; HR, 0.17; 95% CI, 0.04-0.79; *P* = .01). There was also a statistically significant advantage for PSMAiSRT in a small subset of patients who did not receive adjuvant HT during SRT (n = 19 [15%]; HR, 0.00; *P* = .006). Among patients in the experimental arm with a PSA level equal to or greater than the median (n = 28), 17 (61%) had PSMA-PET–positive lesions and received intensified RT. In the experimental cohort, recurrence was observed in 5 patients (8%) who received intensified RT based on a positive PSMA-PET result, as well as in 5 patients (8%) who had a negative PSMA-PET result before starting salvage radiotherapy (SRT). Nine (90%) participants with BCR in the PSMAiSRT study had positive margins and T3-positive disease.

### Toxic Events

Toxic events are summarized in eTable 4 in Supplement 2. Grade 2 or higher RT-related events totaled 60 in the control arm and 59 in the PSMAiSRT arm. Grade 3 or higher events were 3 in the control arm and 6 in the PSMAiSRT arm, with only 1 (grade 3 urethral stenosis requiring surgery) potentially attributable to increased prostate bed RT boost dose of equivalent dose in 2-Gy fractions (α/β 1.4) 76 Gy. Other grade 3 or higher events included cardiac stent placement, lymphedema drainage, and a penile prosthesis for erectile dysfunction.

Overall, there were no differences in EFS for grade 2 or higher seen at 6 months (PSMAiSRT, 17 events vs control, 25 events; *P* = .34) or at median follow up (PSMAiSRT, 34 events vs control, 39 events, *P* = .62). No significant differences were also observed for EFS for grade 3 or higher events at 6 months (HR, 2.01; 95% CI, 0.18-22.17; *P* = .54) or at median follow up (HR, 2.18; 95% CI, 0.54-8.74; *P* = .25). Survival curves displaying EFS comparing toxic events are shown in eFigure 2 in Supplement 2. EFS results are described in eTable 5 in Supplement 2.

### QoL

QoL surveys were completed annually, with a 59% (n = 38) completion rate in the control group and 77% (n = 49) in the experimental arm, due to the lack of follow-up after electronic distribution. Both arms showed no significant MIDs across all domains. Details are in eTable 6 in Supplement 2.

## Discussion

To our knowledge, this is the first prospective randomized clinical trial using PSMA-PET to guide RT intensification with cancer control as the primary end point. Our study demonstrates that the addition of PSMA-PET improved FFS (84% vs 69%; HR, 0.46; 95% CI, 0.22-0.99; *P* = .04) at a median follow-up of 37 months. In addition, PSMAiSRT showed significant improvements in TFS (HR, 0.32; 95% CI, 0.10-1.00; *P* = .04) and eugonadal FFS (HR, 0.45; 95% CI, 0.21-0.96; *P* = .03). The primary end point was based on a strict BCR definition of nadir-positive PSA levels of 0.2 ng/mL or higher after SRT.^20^ Although some clinical trials have used higher thresholds of PSA to define a BCR,^4,5^ a constant rise of PSA is generally observed after it has reached 0.2 ng/mL, thus predicting the use of the next line of therapy and clinical disease progression. Furthermore, this threshold of a PSA level of 0.2 ng/mL or higher is also currently being used widely as a clinical factor to proceed to PSMA-PET imaging to map sites of disease recurrence before proceeding to next-line therapy.^18^

Ongoing or completed trials, including the EMPIRE-1 trial, have shown benefits of using PET imaging, such as 18F-fluciclovine-PET, for patients with BCR after RP to guide SRT.^21^ Contrary to our study, the EMPIRE-1 trial excluded from analysis patients with distant metastases on PET results, which may have led to a selection bias. Second, the radiotracer used in the EMPIRE-1 trial was 18F-fluciclovine, which has been shown to be less performant than PSMA-PET in detecting prostate cancer.^22^ In our study, physicians maintained treatment volumes (eg, pelvis and prostate bed) even without PSMA-PET uptake in pelvic nodes because deescalation of radiotherapy was not permitted, unlike in the EMPIRE-1 clinical trial. In addition, more patients received adjuvant HT during SRT (54 [84%] PSMAiSRT vs 55 [86%] control) than in the EMPIRE-1 clinical trial (30 [38%] experimental vs 28 [35%] control), possibly explaining better FFS outcomes. Another ongoing clinical trial NCT03582774,^11^ has recruited 193 post-RP patients comparing PSMA-PET to conventional imaging including fluciclovine PET, with results pending.

Importantly, before undergoing PSMA-PET, only 19 (30%) in the experimental cohort was intended to receive pelvic RT in comparison to 31 (47%) in the control group, which could have negatively impacted the PSMAiSRT arm because pelvic node RT has been shown to have superior oncologic outcomes.^5^ Nevertheless, PSMA-PET improved FFS by modifying volumes and doses without increasing grade 2 or higher or 3 or higher toxic effects or adversely impacting patient-reported QoL. Baseline characteristics may have also influenced outcomes, with more grade group 5 pathologies (14 [21%] vs 3 [5%]) and positive surgical margins (34 [53%] vs 20 [32%]) in the experimental arm, thereby favoring the control group.^23^ This was reflected in our study, as 9 of patients (90%) in the PSMAiSRT arm who experienced subsequent BCR progression had positive margins at the time of RP. Furthermore, the higher rate of cases with positive resection margins in the experimental group may have influenced the planned pelvic radiotherapy volumes (19 [30%] PSMAisRT vs 31 [47%] control) prior to randomization because radiation oncologists may have chosen to omit pelvic radiotherapy when positive margins were present.

In post-hoc subset analysis, a notable advantage (HR, 0.17; 95% CI, 0.04-0.79; *P* = .01) was found for the PSMAiSRT arm when patients were treated with SRT and PSA levels of 0.3 ng/mL or higher. However, recent consensus to initiate SRT following RP remains at low PSA values of 0.1 to 0.2 ng/mL.^3,4,24^ Furthermore, the difference in treatment-free survival (HR, 0.32; 95% CI, 0.10-1.00; *P* = .04) resulted in more next-line hormonal therapy being initiated in the control group,

Among patients in the experimental cohort, analysis suggests that patients who received intensified radiotherapy due to PSMA-PET–avid lesions had similar outcomes to those with negative PSMA-PET results. Specifically, 5 of 33 patients (15%) with intensified radiotherapy due to positive PSMA-PET results and 5 of 31 patients (16%) with negative PSMA-PET results before initiating SRT experienced subsequent recurrence. This suggests the hypothesis that intensified radiotherapy is effective in eradicating PSMA-PET–positive sites, resulting in outcomes similar to those of patients with negative imaging results.

### Limitations

This randomized clinical trial had some limitations. First, although the median follow-up duration was sufficient to report our primary study end point, it is essential to seek an understanding of impact on longer-term outcomes. Second, the post hoc subset analysis included small sample sizes, limiting the ability to draw strong conclusions from these results. Third, it is known that most patients develop considerable clinical adverse effects after completing RT but tend to recuperate after 12 months of treatment. For this reason, the annual QoL analysis in our study may have underestimated the transient effects of RT on the 5 evaluated domains. Lastly, as this trial was a pragmatic registry-based design, specific criteria for defining standard RT volumes (eg, prostate bed alone vs whole-pelvis RT) and prescribing hormonal therapy were intentionally omitted, reflecting care as usual. However, the lack of specific follow-up parameters such as testosterone monitoring resulted in a subset of patients receiving hormonal therapy (5 [9%] in the PSMAiSRT group and 7 [13%] in the control group) lacking testosterone data, which may have compromised the outcomes of eugonadal FFS.

## Conclusion

To our knowledge, this is the first randomized clinical trial demonstrating an isotoxic improvement in prostate cancer control outcomes with intensification of SRT informed by PSMA-PET in men who present with BCR after RP, in a modern cohort with prevalent use of adjuvant HT. Although this imaging-guided technique has demonstrated advantages, it seems most effectively applied to patients undergoing SRT with a PSA level of 0.3 ng/mL or higher. Given its impact, PSMA-PET should be performed to guide SRT for patients presenting with a BCR after RP. A subsequent phase 3 randomized clinical trial (NCT04557501) has completed accrual and will shed further light on those patients who benefit most from PSMAiSRT.

## Notes

Supplement 1. Trial Protocol

Supplement 2. eTable 1. Testosterone levels at median follow-up eTable 2. Duration of hormonal therapy at salvage radiotherapy eTable 3. Sites of failure at biochemical progression eFigure 1. Kaplan Meier curves displaying FFS for patients with pre-SRT PSA ≥ 0.3 ng/mL and for patients treated without adjuvant HT eTable 4. Frequency of grade 2+ and grade 3+ adverse events attributable to RT eTable 5. Patient-Based Cumulative Incidence of Grade 2+ and Grade 3+ Adverse Events for Event-Free Survival Analysis Figure 2. Freedom From First CTCAEv.5 Event eTable 6. MID events per EPIC-CP domain at different time points

Supplement 3. Data Sharing Statement

## References

[1] Freedland SJ, Humphreys EB, Mangold LA, . Risk of prostate cancer-specific mortality following biochemical recurrence after radical prostatectomy. JAMA. 2005;294(4):433-439. doi:10.1001/jama.294.4.433 16046649

[2] Lee E, Singh T, Han M, . Early initiation of salvage radiotherapy is associated with improved metastasis-free survival in patients with relapsed prostate cancer following prostatectomy. JCO. 2022;40(6)(suppl):262-262. doi:10.1200/JCO.2022.40.6_suppl.262 36316967

[3] Kneebone A, Fraser-Browne C, Duchesne GM, . Adjuvant radiotherapy versus early salvage radiotherapy following radical prostatectomy (TROG 08.03/ANZUP RAVES): a randomised, controlled, phase 3, non-inferiority trial. Lancet Oncol. 2020;21(10):1331-1340. doi:10.1016/S1470-2045(20)30456-3 33002437

[4] Parker CC, Clarke NW, Cook AD, . Timing of radiotherapy after radical prostatectomy (RADICALS-RT): a randomised, controlled phase 3 trial. Lancet. 2020;396(10260):1413-1421. doi:10.1016/S0140-6736(20)31553-1 33002429 PMC7616947

[5] Pollack A, Karrison TG, Balogh AG, . The addition of androgen deprivation therapy and pelvic lymph node treatment to prostate bed salvage radiotherapy (NRG Oncology/RTOG 0534 SPPORT): an international, multicentre, randomised phase 3 trial. Lancet. 2022;399(10338):1886-1901. doi:10.1016/S0140-6736(21)01790-6 35569466 PMC9819649

[6] Yuminaga Y, Rothe C, Kam J, . ^68^Ga-PSMA PET/CT versus CT and bone scan for investigation of PSA failure post radical prostatectomy. Asian J Urol. 2021;8(2):170-175. doi:10.1016/j.ajur.2020.02.001 33996472 PMC8099644

[7] Giesel FL, Knorr K, Spohn F, . Detection efficacy of ^18^F-PSMA-1007 PET/CT in 251 patients with biochemical recurrence of prostate cancer after radical prostatectomy. J Nucl Med. 2019;60(3):362-368. doi:10.2967/jnumed.118.212233 30042163 PMC6424235

[8] Calais J, Czernin J, Cao M, . ^68^Ga-PSMA-11 PET/CT mapping of prostate cancer biochemical recurrence after radical prostatectomy in 270 patients with a PSA level of less than 1.0 ng/mL: impact on salvage radiotherapy planning. J Nucl Med. 2018;59(2):230-237. doi:10.2967/jnumed.117.201749 29123013 PMC5807533

[9] Ng M, Guerrieri M, Wong LM, . Changes in management after ^18^F-DCFPyL PSMA PET in patients undergoing postprostatectomy radiotherapy, with early biochemical response outcomes. J Nucl Med. 2022;63(9):1343-1348. doi:10.2967/jnumed.121.263521 35058320 PMC9454460

[10] Sood A, Kishan AU, Evans CP, . The impact of positron emission tomography imaging and tumor molecular profiling on risk stratification, treatment choice, and oncological outcomes of patients with primary or relapsed prostate cancer: an international collaborative review of the existing literature. Eur Urol Oncol. 2024;7(1):27-43. Published online July 8, 2023. doi:10.1016/j.euo.2023.06.002 37423774

[11] Armstrong WR, Kishan AU, Booker KM, . Impact of prostate-specific membrane antigen positron emission tomography/computed tomography on prostate cancer salvage radiotherapy management: results from a prospective multicenter randomized phase 3 trial (PSMA-SRT NCT03582774). Eur Urol. 2024;86(1):52-60. doi:10.1016/j.eururo.2024.01.012 38290964 PMC12882065

[12] Petit C, Delouya G, Taussky D, . PSMA-PET/CT-guided intensification of radiation therapy for prostate cancer (PSMAgRT): findings of detection rate, effect on cancer management, and early toxicity from a phase 2 randomized controlled trial. Int J Radiat Oncol Biol Phys. 2023;116(4):779-787. doi:10.1016/j.ijrobp.2022.12.055 36639035

[13] Schulz KF, Altman DG, Moher D; CONSORT Group. CONSORT 2010 statement: updated guidelines for reporting parallel group randomised trials. BMC Med. 2010;8:18. doi:10.1186/1741-7015-8-18 20334633 PMC2860339

[14] Dornan M, Simard JM, Leblond A. Simplified and robust one-step radiosynthesis of [18F]DCFPyL via direct radiofluorination and cartridge-based purification. J Label Compd Radiopharm. 2018;61(10):757-763. doi:10.1002/jlcr.3632 29722070

[15] Stephenson AJ, Scardino PT, Kattan MW, . Predicting the outcome of salvage radiation therapy for recurrent prostate cancer after radical prostatectomy. J Clin Oncol. 2007;25(15):2035-2041. doi:10.1200/JCO.2006.08.9607 17513807 PMC2670394

[16] Rowe SP, Macura KJ, Mena E, . PSMA-based [(18)F]DCFPyL PET/CT is superior to conventional imaging for lesion detection in patients with metastatic prostate cancer. Mol Imaging Biol. 2016;18(3):411-419. doi:10.1007/s11307-016-0957-6 27080322 PMC5261857

[17] Ménard C, Young S, Zukotynski K, . PSMA PET/CT guided intensification of therapy in patients at risk of advanced prostate cancer (PATRON): a pragmatic phase III randomized controlled trial. BMC Cancer. 2022;22(1):251. doi:10.1186/s12885-022-09283-z 35260100 PMC8902723

[18] De Visschere PJL, Standaert C, Fütterer JJ, . A systematic review on the role of imaging in early recurrent prostate cancer. Eur Urol Oncol. 2019;2(1):47-76. doi:10.1016/j.euo.2018.09.010 30929846

[19] Chipman JJ, Sanda MG, Dunn RL, ; PROST-QA Consortium. Measuring and predicting prostate cancer related quality of life changes using EPIC for clinical practice. J Urol. 2014;191(3):638-645. doi:10.1016/j.juro.2013.09.040 24076307 PMC5006995

[20] Jackson WC, Suresh K, Tumati V, . Impact of biochemical failure after salvage radiation therapy on prostate cancer-specific mortality: competition between age and time to biochemical failure. Eur Urol Oncol. 2018;1(4):276-282. doi:10.1016/j.euo.2018.04.014 31100248

[21] Jani AB, Schreibmann E, Goyal S, . ^18^F-fluciclovine-PET/CT imaging versus conventional imaging alone to guide postprostatectomy salvage radiotherapy for prostate cancer (EMPIRE-1): a single centre, open-label, phase 2/3 randomised controlled trial. Lancet. 2021;397(10288):1895-1904. doi:10.1016/S0140-6736(21)00581-X 33971152 PMC8279109

[22] Olivier P, Giraudet AL, Skanjeti A, . Phase III study of ^18^F-PSMA-1007 versus ^18^F-fluorocholine PET/CT for localization of prostate cancer biochemical recurrence: a prospective, randomized, crossover multicenter study. J Nucl Med. 2023;64(4):579-585. doi:10.2967/jnumed.122.264743 36418170 PMC10071780

[23] Wright JL, Dalkin BL, True LD, . Positive surgical margins at radical prostatectomy predict prostate cancer specific mortality. J Urol. 2010;183(6):2213-2218. doi:10.1016/j.juro.2010.02.017 20399459 PMC2903223

[24] Sargos P, Chabaud S, Latorzeff I, . Adjuvant radiotherapy versus early salvage radiotherapy plus short-term androgen deprivation therapy in men with localised prostate cancer after radical prostatectomy (GETUG-AFU 17): a randomised, phase 3 trial. Lancet Oncol. 2020;21(10):1341-1352. doi:10.1016/S1470-2045(20)30454-X 33002438

