# Supplementary material for: Prostate-Specific Membrane Antigen PET-Guided Intensification of Salvage Radiotherapy After Radical Prostatectomy: A Phase 2 Randomized Clinical Trial
Source: JAMA Oncol. 2025 Oct 2;11(12):1431–8. doi: 10.1001/jamaoncol.2025.3746 (PMC12581501; doi:10.1001/jamaoncol.2025.3746)
Supplement: Supplement 1. — Trial Protocol [file jamaoncol-e253746-s001.pdf]

Principal investigator:

Cynthia Ménard, MD, radiation oncologist, CHUM

Co-Investigators (CHUM):

Guila Delouya, MD, FRCPC, radiation oncologist, CHUM

Fred Saad, MD, FRCPC, uro-oncologist, CHUM

Daniel Juneau, MD, FRCPC, nuclear medicine, CHUM

Jean DaSilva, PhD, radiochemistry, CRCHUM

Protocol Summary

**Title**	PSMA-PET Guided Radiotherapy in Patients with High-Risk, Recurrent, or Oligometastatic Prostate Cancer (PERa GU 17.1)
**Population**	Patients with high-risk, recurrent, or oligometastatic prostate cancer planned for definitive radiotherapy.
**Number of Experimental Subjects**	n=130
**Number of Sites**	Two- CHUM, CSSSL,
**Duration of Patient Study Participation**	5 years
**Objectives**	**Primary Objective**<br>1. To determine if PSMA-PET/CT guided RT is superior to standard RT as measured by improved failure-free survival.<br><br>**Secondary Objectives**<br>2. To determine acute and delayed toxicities.<br>3. To determine the rates of biochemical, radiological, local, regional, and distant failures, and time to next therapy.<br>4. To measure prostate-cancer specific and overall survival.<br>5. To measure health-related quality of life.<br>6. To measure the detection yield of PSMA PET/CT imaging.
**Primary Hypothesis**	PSMA-PET/CT image-guided radiotherapy (IGRT) improves failure-free survival compared conventional IGRT in patients at high-risk of metastases.
**Estimated Time to Complete Enrollment**	Patients will be enrolled over 3 years.

1.0 Introduction

Prostate cancer (PCa) is the most common non-skin malignancy and the third leading cause of cancer death in North American men.[1] The accurately mapped metastatic state is a necessary prerequisite to guiding treatment in practice and in clinical trials.[2, 3] Imaging biomarkers (BMs) can provide information on disease volume and distribution, prognosis, changes in biologic behavior, therapy-induced changes (both responders and non-responders), durations of response, emergence of treatment resistance, and the host reaction to the therapies. The impact of advanced imaging is expected to be heightened in the context of novel therapeutics as well as in image-guided therapies, such as radiotherapy and stereotactic ablative radiotherapy (SABR).

Of particular relevance to metastatic prostate cancer is the emergence of a promising imaging technique involving new prostate specific membrane antigen (PSMA) positron emission tomography (PET) tracers. This approach has demonstrated higher sensitivity in detecting metastases, prior to and during therapy, than current imaging standard of care (CT and bone scan), and is not widely clinically available outside of the research realm in North America.

Positron emission tomography / computer tomography (PET/CT) is a nuclear medicine diagnostic imaging procedure based on the measurement of positron emission from radiolabeled tracer molecules *in vivo*. PSMA is a homodimeric type II membrane metalloenzyme that functions as a glutamate carboxypeptidase/folate hydrolase and is overexpressed in PCa. PSMA is expressed in the vast majority of PCa tissue specimens and its degree of expression correlates with a number of important metrics of PCa tumor aggressiveness including Gleason score, propensity to metastasize and the development of castration resistance.[4]

[18F]DCFPyL is a promising high-sensitivity second generation PSMA-targeted urea-based PET probe. Studies employing second-generation PSMA PET/CT imaging in men with biochemical progression after definitive therapy suggest detection of metastases in over 60% of men imaged.[3] In fact, PSMA-based PET has so far proven to have higher sensitivity than any other modality for localization of the site of recurrence. Applications that show promise and require further investigation include the characterization and risk stratification of primary PCa, complete staging of metastatic PCa to allow for PSMA-targeted radiotherapy and improved identification of patients with oligometastatic disease. One of the main advantage of this new PET tracer is that it is labeled with Fluor-18 instead of Gallium-68, which is the most commonly used ligand

in other PSMA targeted probes. The radionuclide F-18 has several physical advantages compared to Ga-68, including lower energy and shorter positron range providing higher image resolution, longer half-life (110min vs. 68min) enabling multiple scans from a single formulation, and shipment to other sites without radiochemistry/cyclotron capability.

Here we hypothesize that definitive RT informed by PSMA-PET findings will lead to improved cancer control outcomes compared with RT guided by conventional staging only. We will utilize a cmRCT design, where eligible patients will be randomly selected for PSMA-PET/CT imaging prior to RT and approached for this investigational study. This study will therefore be conducted in companion to PERA (Partnership initiative for the Evaluation of technological innovation in Radiotherapy, CHUM CER 17.032).

2.0 Objectives

2.1 Primary objective

2.1.1 To determine if PSMA-PET/CT guided RT is superior to standard RT as measured by improved failure free survival.

2.2 Secondary objectives

2.2.1 To determine acute and delayed toxicities.

2.2.2 To determine the rates of biochemical, radiological, local, regional, and distant failures, and time to next line therapy.

2.2.3 To measure prostate cancer specific and overall survival.

2.2.4 To measure health-related quality of life.

2.2.5 To measure the detection yield of PSMA PET/CT imaging.

3.0 Eligibility Assessments

3.1 Inclusion criteria

3.1.1 Enrolled in PERa registry (CHUM CER 17.0.32), consented to contact for investigational trials, consented to serve as control, and randomly selected to be offered the experimental intervention.

3.1.2 Histological diagnosis of prostate cancer planned for definitive-intent radiotherapy.

3.1.3 ECOG 0-1

3.1.4 Charlson Cormobidity Index ≤ 4

3.1.5 High-risk of distant metastases as defined by any of:

3.1.5.1 Oligometastases (≤5) (regional or distant) identified on conventional staging, with ≤ 3 metastasis in any non-bone organ. For a spine metastasis, direct involvement of adjacent spinal segments would still be considered as “one” tumour. For nodal metastases, more than one involved lymph node in the same ipsilateral nodal region/chain would still count as “one” tumour. Defined nodal regions for this protocol include inguinal, external iliac, internal iliac, common iliac, retroperitoneal, hilar/mediastinal, anterior cervical, posterior cervical, and axillary. Metastases in all other organs that are within 1cm of each other will be considered as “one” tumour.

3.1.5.2 Subjects with newly diagnosed high-risk (NCCN) localized prostate cancer and CAPRA score 6-10.

3.1.5.3 Subjects with a prior history of treated prostate cancer

- Post-radical prostatectomy and biochemical failure (> 0.1ng/ml)
- Post-curative radiotherapy and biochemical failure (Phoenix-RT)

3.2 Exclusion Criteria

3.2.1 Prior androgen deprivation therapy terminated < 12 months prior to enrollment.

3.2.2 Prior or planned PET scan.

4.0 Enrollment procedures

4.1 Confirmation of patient`s eligibility

4.2 Enrollment will occur after consent is completed.

4.3 Study Registration

4.3.1 Registration will occur only after pre-treatment evaluations and informed consent are completed.

6.0 Study Implementation

6.1 Study Design

6.1.1 Nested within the Partnership Initiative for the Evaluation of Technological Innovation in Radiotherapy (PERA - NCT03378856) prospective cohort, we will perform a Randomized Controlled Trial (RCT) comparing PSMA-guided radiotherapy (PSMAgRT) to controls.

When entering the PERA cohort, consent is asked for the use of data and for being approached for future research. Random selection of eligible patients to this trial were offered PSMAgRT. Outcomes will be compared with eligible patients randomized to a control arm and receiving usual care.

6.2 Procedures

6.2.1 PSMA -PET radiotracer provided by either: CRCHUM or PROGENICS Pharmaceuticals Inc. Use of this radiotracer in humans at the CHUM will be permitted following approval of this Positron-emitting Radiopharmaceutical for use in a CTA from Health Canada.

6.2.2 PSMA -PET/CT scanning

6.2.2.1 To be performed within 4 weeks of registration (or when PSA > 0.1ng/ml for patient post RP)

6.2.2.2 [18F]-DCFPyL PET/CT studies will be performed on hybrid PET/CT scanners which combine a dedicated, full-ring PET scanner with a multi-slice spiral CT scanner. Patients should fast for at least 4 hours to facilitate transit of oral contrast into the distal small bowel. A volume of 200-400 ml of barium sulfate oral contrast can be administered and 9 ± 1 mCi (333 ± 37 MBq, calculated and decay-corrected) of [18F]-DCFPyL is injected by slow intravenous push followed by saline flush. 90 minutes (+/- 30 minutes)

following [18F]-DCFPyL injection, CT and PET images are consecutively acquired from the base of the skull to mid-thighs. Additional images of the skull, lower extremities or upper extremities might be acquired if there is a clinical suspicion of lesion at these sites. Repeat imaging of equivocal foci can be obtained up to 180 minutes post-IV. Optionally, Lasix 10-40 mg IV may be administered 30-60 minutes prior to primary or repeat imaging to help clear urinary bladder activity.

6.2.2.3 For the CT scan portion of the study, the following are suggested, (slight variation based on clinical sites' clinical routine is acceptable): 120-140 kVp, 60–140 mA (depending on the body weight or noise index), a rotation time of 0.8 s, a pitch of 1.75:1, and a detector row configuration using 0.625 mm thick detectors. For the PET portion of the study, a 3D acquisition is performed and images are acquired using 2-5 min per bed position (depending on the body weight and PET detector crystal composition) and 5 to 6 bed positions are used (depending on the patient's height). Non-stop-motion PET is also acceptable if the PET scanner has this feature. Time-of-flight imaging is encouraged if the PET scanner has this feature. PET attenuation-corrected, PET non-attenuation-corrected, CT, and fused images are available for review in the transaxial, coronal, and sagittal planes with an ordered subset expectation maximization (OSEM) or similar iterative algorithm for the PET images.

6.2.2.4 The uptake of the lesions will be measured with SUVmax. SUVmax will be calculated using the usual formula with computations that also consider the post-injection residual activity. Patients will be weighed in the imaging department. Hepatic and blood pool SUV will be determined for the computation of tumor-to-liver and tumor-to-blood ratios.

6.2.2.5 Experienced central readers from each institution will adjudicate by consensus for each newly detected lesion beyond standard-care imaging. Disagreements between two experienced readers will be adjudicated by a tie-breaking third vote of a

separate nuclear medicine physician. Central review may result in recommendation of MRI and/or biopsy of suspicious lesions.

6.2.2.6 Reports will be provided to treating physicians within 3 weeks of imaging.

6.2.2.7 Images will be provided if requested by treating physician to assist in treatment planning.

6.1.3 Confirmation MRI: In cases where PSMA -PET/CT findings are uncertain, an MRI of the questioned lesion site may be performed at the discretion of the treating team.

6.1.4 Radiotherapy

6.1.4.1 PSMA -PET/CT imaging – no additional lesions detected: If no additional lesions are detection, radiotherapy to proceed as planned per standard care.

6.1.4.2 PSMA-PET/CT imaging – consistent with oligometastases (1- 5 lesions): ALL oligometastatic lesions must be treated with definitive radiotherapy. (see Guide – Appendix 1)

6.1.4.3 PSMA-PET/CT imaging – consistent with widely metastatic disease (>5 lesions): Treatment of all detected disease with radiotherapy is not recommended. Physicians may alter their radiotherapy plan as their discretion. However treatment of the primary disease is encouraged.

6.1.4.4 RT summary to be provided (Impact of PSMA, Date completed, Dose, Fractionation, Sites treated)

6.1.5 Hormonal therapy

6.1.5.1 At the discretion of the treating physician. However a change in hormonal therapy is discouraged.

6.1.6 Follow-up

6.1.6.1 Per standard care practice / PERa, at least annually with PSA and EPIC questionnaires.

6.1.6.2 Biochemical failures (Phoenix) to be investigated per standard care.

6.2 Withdrawal Criteria

Patients may be taken off study prior to the completion of study related procedures for the following reasons:

6.2.1 Patient withdraws consent for participation.

6.2.2 It is deemed in the patient`s best interest as determined by the attending/PI.

6.2.3 Serious protocol violation as determined by the PI

6.2.4 Development of a concurrent serious medical condition during the studies or treatments precluding completion of study procedures.

6.2.4 Completion of per-study follow-up (5 years after radiotherapy)

7.0 Data collection and Confidentiality

7.1. Hard Copies

6.1.1.1 Consent forms

7.2 Electronic Data

7.2.1 Medical records (Eg. EMR, OACIS, MOSAIC)

7.2.2 Imaging Data (Eg. Clinical PACS, Research PACS), including anonymized electronic submission for the research access use of 18F-DCFPyL imaging.

7.2.3 Research Data (Eg. Excel spreadsheet, Research PACS, Nuclear Medicine Reports, CASTOR EDC)

7.3 Confidentiality

7.3.1 In all research records, subjects will be identified by enrollment number.

7.3.2 Research data will be under secure password, or kept under lock and key in the research office.

7.3.3 No records bearing patients identification will be provided to anyone outside of the institution except regulatory agencies.

7.3.4 Patients will not be identifiable as individuals in any publication or presentation that may result from this study.

7.4 Data Safety and monitoring plan

7.4.1 PI will assume primary responsibility for monitoring the progress of the trial and the safety of participants.

7.4.2 Data will be sumitted to the REC annually for continuing review and at the completion of the study per institutional guidelines.

8.0 Study Endpoints

8.1 Failure-free survival, defined as time to biochemical failure (Phoenix definition, Roach 2006, or >0.2ng/ml for post-RP strata), local failure, regional failure, distant metastases, initiation of the next line of therapy, or death from any cause. (measured from end of RT) Event-free patients are censored at their last known follow-up date.

8.2 CTCAE v5.0: Attributable Gr2+ toxicities.

8.3 Rates of biochemical failure, local failure, regional failure, distant failure, and radiological failure.

8.4 Disease specific and overall survival

8.5 Health related quality of life: EPIC-CP, FACT-P, IPSS

8.6 Detection yield of PSMA-PET

9.0 Human subjects protection

9.1 Evaluation of Risks/Discomforts

9.1.1 <u>Risk of PSMA -PET/CT imaging:</u> There is extensive clinical experience using ^{18}F-lableled radiotracers, especially flurodeoxyglucose (FDG). This experience has demonstrated the safety of PET imaging, which is now used routinely for diagnostic, radiation treatment planning and research purposes worldwide. There are no risks related to the PET imaging acquisition itself, potential risks are probe-specific.

Radiopharmaceuticals are generally not associated with side effects. Allergic reactions are excessively rare. Pharmacokinetic

and dosimetric characterization of PSMA-PET has been previously performed in humans[34]. Rapid and nearly static biodistribution after 20-30 minutes has been observed for [^{18}F]DCFPyL, with negligible blood/serum/RBC activity measured 25 minutes after injection . The effective (whole body) dose for 370MBq (10mCi) [^{18}F]DCFPyL injection is approximately 0.0165 mSv/MBq. This dose is below the 0.03mSv/MBq upper limit recommendation by the FDA for diagnostic tests, and corresponds roughly to 2.5 additional years of background exposure. The dose-critical organs doses are: kidneys (0.0896 mGy/MBq), bladder wall (0.0873 mGy/MBq), submandibular glands (0.0418 mGy/MBq), and liver (0.0420 mGy/MBq). Preliminary studies have showed high rates of uptake in tumours of patients with prostate and renal cancer[34, 39], and increased performance compared to other PSMA-targeted probes[40]. No documented serious adverse events (SAE) considered related to 18F-DCFPyL have been recorded in the trials using [^{18}F]DCFPyL, and its use has been previously approved for investigational purposes by Health Canada.

During PSMA -PET/CT scanning, patients are exposed to a small dose of radiation (10-12mSv). This dose is not associated with any risk in the context of patients receiving radiotherapy.

9.1.2 Risk of IV injection: Use of imaging contrast/probe agents during intravenous injection will involve minimal discomfort to the patient and can occasionally result in mild soreness or bruising. This may rarely lead to superficial thrombophlebitis in less than 5% of patients.

9.1.3 Risk of radiation toxicity: Radiation treatment to oligometastatic sites identified on PSMA -PET/CT may lead to side-effects. Dose objective and normal tissue exposure constraints will respect standard care practice; therefore the risks are equivalent to standard-care definitive radiotherapy to those sites.

Acute side effects are expected to be low (<10%), they include fatigue, skin reactions, and side-effects specific to the areas being treated. In rare instances, exposure to high doses of radiotherapy may result in late severe adverse effects such as: myelitis,

neurological deficits, proctitis, bowel symptoms and/or perforation. Nonetheless, considering the expected low burden (and volumes) of disease to be treated, the study has been designed to respect standard-care dose-volume limits to organs at risk, while maintaining a definitive dose to the tumor. In this manner, the risk of severe late adverse events is expected to be very low (<5%).

9.2 Adverse Event Documentation & Reporting

9.1 Adverse event criteria

9.1.1 Toxicities will be evaluated and graded using CTCAE v5.0

9.1.2 Patients will be monitored for potential PSMA -PET-related or RT-related adverse events.

9.1.3 All adverse events of grade 2 or higher, whether serious or not, will be recorded if attributable to PSMA -PET/CT imaging or change in RT.

9.1.4 Any adverse event attributable to study intervention will be followed by the investigator until the event is resolved, the subject is lost to follow-up, the symptoms resolved, or the adverse event is found to be unrelated to the study.

9.1.5 An adverse event (AE) is any untoward, undesired, unplanned medical occurrence in a participant and does not necessarily have a causal relationship with the study intervention. An adverse event can therefore be any unfavourable and unintended sign (including an abnormal laboratory finding or physiological observations), symptom or disease temporally associated with the use of a medical treatment or procedure that may or may not be considered related to the medical treatment or procedure. Any symptom, sign, illness, or experience that develops or worsens in severity during the course of the study, including intercurrent illnesses or injuries, should be regarded as an adverse event.

9.1.6 Adverse events are classified as serious or non-serious. A Serious Adverse Event (SAE) is defined as any untoward medical occurrence/AE that:

9.1.6.1 Results in death;

9.1.6.2 Is life-threatening (refers to any adverse event that places the subject at immediate risk of death from the event as it occurred; life-threatening event does not include an event that, had it occurred in a more severe form, might have caused death, but as it actually occurred, did not create an immediate risk of death);

9.1.6.3 Requires in-patient hospitalization and/or prolongation of an existing hospitalization (hospitalization refers to an overnight admission). Emergency room visits are not considered serious until one of the above criteria is met. Any elective hospitalization for a pre-existing condition that has not worsened does not constitute an SAE;

9.1.6.4 Results in persistent or significant disability or incapacity (substantial disruption in a person's ability to conduct normal daily living activities); a congenital anomaly or birth defect; or other medically important event.

9.3 Risk/Benefit Analysis

Patients may benefit from participation in this study. Results will be provided to the treating physician, and may lead to a change in treatment of prostate cancer. Although such a change would be expected to benefit patients, it is also possible that a change in treatment will lead to side effects.

10.0 ETHICS

10.1 The study will be submitted for initial and ongoing annual REB review. Study related procedures will not commence prior to receipt of REB approval.

10.2 Any amendment to the study will be submitted for review by the REB before any changes are implemented unless required to eliminate immediate hazard to the study participants.

10.3 Data derived from this study may be reported in scientific publications. Patients will not be indicated by name.

10.4 Sample size determination

This study will employ a cmRCT design to determine the potential benefit of PSMA-PET guided radiotherapy in patient with high-risk of micro/oligo

metastatic disease. Patients will be randomly selected (1:1) for this intervention from the PERA study if they provide consent and meet eligibility criteria. The study will be designed with an alpha=0.1 and 80% power (as recommended for phase II randomized trials) for the primary outcomes. Patients will be stratified according to institution and category at time of enrollment (oligometastatic - estimated 15% of cohort) vs. high-risk localized – estimated 35% of cohort, vs. recurrent post RT – estimated 5% of cohort vs. recurrent post RP – estimated 45% of cohort).

Literature suggests that PSMA PET/CT can increase the diagnostic yield (sensitivity) for bone metastases by 10-15% compared with standard staging (approximately 85% to 99%), and for lymph node metastases by 50% (25% to 75%), resulting in over 60% of patient with high-risk of metastases demonstrating new identifiable lesions on PSMA PET. Adjusting for the proportion surviving free of failure at 5 years within the strata (0.05, 0.6, 0.3, and 0.4, respectively), a total of 130 patients receiving a PSMA -PET/CT evaluation (and 130 control) will be required to demonstrate benefit over standard care ($\alpha=0.1$, $\beta=0.8$, one-tailed, HR 1.4).

10.5 Interim analysis

2.2.6 An interim analysis will be conducted upon the completion of accrual to assess the rate of new lesion detection and its impact on prostate cancer management.

2.2.7 An interim analysis for the primary endpoint will be conducted when 25% of randomized patients have experienced a failure event within a stratified cohort. The purpose of this analysis is to guide the design of a subsequent phase III trial, including which strata should be included in a next phase clinical trial. The efficacy criterion for the interim analysis will be a two-sided p-value threshold of 0.05 for each individual strata.

11.0 DATA HANDLING AND RECORD KEEPING

Data will be recorded and stored using the following sources:

11.1 Medical records (OASIS, MOSAIC): clinical data
11.2 PACS: imaging data

All records and documents pertaining to the study will be retained by the study trial site for at least 25 years from the completion of the study, and will be available for inspection by Health Canada, institutional representatives (REB, Quality Assurance) and other regulatory bodies.

Coded imaging data may also be shared with industry or academic collaborators outside the institution. The details of this data exchange will be described in separate Data Transfer Agreements (DTAs) between institution and these parties.

Data will not be shared with any external collaborator except the appropriate agreements are in place. Hard copy study files will be kept secured. All computerized files will be password protected and no laptop will be used. Coded research data will be stored on research servers.

Diffusion - aggregate disclosure to study participants and presentation of results at conferences and scientific publications. Participants will not be identifiable in any knowledge transfer.

12.0 References

1. *Canadian Cancer Statistics.* 2015.
2. Halabi, S., et al., *Meta-Analysis Evaluating the Impact of Site of Metastasis on Overall Survival in Men With Castration-Resistant Prostate Cancer.* J Clin Oncol, 2016. **34**(14): p. 1652-9.
3. Evans, C.P., et al., *The PREVAIL Study: Primary Outcomes by Site and Extent of Baseline Disease for Enzalutamide-treated Men with Chemotherapy-naive Metastatic Castration-resistant Prostate Cancer.* Eur Urol, 2016.
4. Evans, M.J., et al., *Noninvasive measurement of androgen receptor signaling with a positron-emitting radiopharmaceutical that targets prostate-specific membrane antigen.* Proc Natl Acad Sci U S A, 2011. **108**(23): p. 9578-82.
5. Banerjee, S.R., et al., *Synthesis and evaluation of technetium-99m- and rhenium-labeled inhibitors of the prostate-specific membrane antigen (PSMA).* J Med Chem, 2008. **51**(15): p. 4504-17.
6. Olberg, D.E., et al., *One step radiosynthesis of 6-[(18)F]fluoronicotinic acid 2,3,5,6-tetrafluorophenyl ester ([(18)F]F-Py-TFP): a new prosthetic group for efficient labeling of biomolecules with fluorine-18.* J Med Chem, 2010. **53**(4): p. 1732-40.
7. Chen, Y., et al., *2-(3-{1-Carboxy-5-[(6-[18F]fluoro-pyridine-3-carbonyl)-amino]-pentyl}-ureido)-pen tanedioic acid, [18F]DCFPyL, a PSMA-based PET imaging agent for prostate cancer.* Clin Cancer Res, 2011. **17**(24): p. 7645-53.

Appendix 1: Guide – Definitive Radiotherapy to Oligometastatic Prostate Cancer

The following doses are meant as a guide. For SABR, it is strongly recommended that patients be referred to a center of expertise for treatment.

Recommended Dose/Fractionation of Definitive RT

- Prostate: EQD2 (alpha/beta=3)=72Gy or more
- Involved lymph nodes: 35-50 Gy in 5 fractions, or a simultaneous integrated boost to pelvic RT (EQD2=66-70Gy, alpha/beta=3)
- Bone metastases: 30-40 Gy/5 fractions, or a simultaneous integrated boost to pelvic RT (EQD2=66-70Gy, alpha/beta=3)
- Lung Metastases (peripheral) : 48-60 Gy / 4 fractions OR 54-60 Gy / 3 fractions
- Lung Metastases (central): 50Gy/5 fractions or 60Gy/8 fractions
- Liver Metastases: 30-60 Gy in 3-5 fractions
- Adrenal/spleen metastases: 35-50Gy in 5 fractions
- Soft tissue metastases not otherwise classifiable: 35-50 Gy in 5 fractions.
