# Supplementary material for: Prostate-Specific Membrane Antigen PET-Guided Intensification of Salvage Radiotherapy After Radical Prostatectomy: A Phase 2 Randomized Clinical Trial
Source: JAMA Oncol. 2025 Oct 2;11(12):1431–8. doi: 10.1001/jamaoncol.2025.3746 (PMC12581501; doi:10.1001/jamaoncol.2025.3746)
Supplement: Supplement 2. — eTable 1. Testosterone levels at median follow-up eTable 2. Duration of hormonal therapy at salvage radiotherapy eTable 3. Sites of failure at biochemical progression eFigure 1. Kaplan Meier curves displaying FFS for patients with pre-SRT PSA ≥ 0.3 ng/mL and for patients treated without adjuvant HT eTable 4. Frequency of grade 2+ and grade 3+ adverse events attributable to RT eTable 5. Patient-Based Cumulative Incidence of Grade 2+ and Grade 3+ Adverse Events for Event-Free Survival Analysis Figure 2. Freedom From First CTCAEv.5 Event eTable 6. MID events per EPIC-CP domain at different time points [file jamaoncol-e253746-s002.pdf]

Supplemental Online Content

Belliveau C, Saad F, Duplan D, et al. Prostate-specific membrane antigen PET-guided intensification of salvage radiotherapy after radical prostatectomy: a phase 2 randomized clinical trial. *JAMA Oncol*. Published online October 2, 2025.
doi:10.1001/jamaoncol.2025.3746

eTable 1. Testosterone levels at median follow-up
eTable 2. Duration of hormonal therapy at salvage radiotherapy
eTable 3. Sites of failure at biochemical progression
eFigure 1. Kaplan Meier curves displaying FFS for patients with pre-SRT PSA ≥ 0.3 ng/mL and for patients treated without adjuvant HT
eTable 4. Frequency of grade 2+ and grade 3+ adverse events attributable to RT
eTable 5. Patient-Based Cumulative Incidence of Grade 2+ and Grade 3+ Adverse Events for Event-Free Survival Analysis
Figure 2. Freedom From First CTCAEv.5 Event
eTable 6. MID events per EPIC-CP domain at different time points

This supplemental material has been provided by the authors to give readers additional information about their work.

© 2025 American Medical Association. All rights reserved, including those for text and data mining, AI training, and similar technologies.

eTable 1 . Testosterone levels at median follow-up.

	**PSMAiSRT (n=54)**	**Control (n=55)**
Eugonadal at median follow-up (Testosterone ≥5.2 ng/L)	43 (80%)	43 (78%)
Castrated at median follow-up (Testosterone <5.2 ng/L)	6 (11%)	5 (9%)
No testosterone on follow-up	5 (9%)	7 (13%)

eTable 2. Duration of hormonal therapy at salvage radiotherapy.

	**PSMAiSRT**	**Control**
**Hormonotherapy**	**54 (84%)**	**55 (86%)**
intermittent	0	0
≤ 6 months	48 (89%)	50 (91%)
]6-18[months	2 (4%)	3 (5%)
18 months	0	0
> 18 months	4 (7%)	2 (4%)
continuous	0	0
ARPI	0	0

ARPI : Androgen receptor pathway inhibitors

eTable 3. Sites of failure at biochemical progression

Failure events	PSMAiSRT (n=10)	Control (n=20)
**Locoregional**	1 (10%)	5 (25%)
**Distant**	5 (50%)	7 (35%)
**No lesion found to date**	4 (40%)	8 (40%)
**Negative PSMA or HT initiated**	1	6
**PSMA pending**	1	1
**Death from unrelated causes**	1	1
**Study withdrawal**	1	0

eFigure 1. Kaplan Meier curves displaying FFS for patients with pre-SRT PSA ≥ 0.3 ng/mL and for patients treated without adjuvant HT

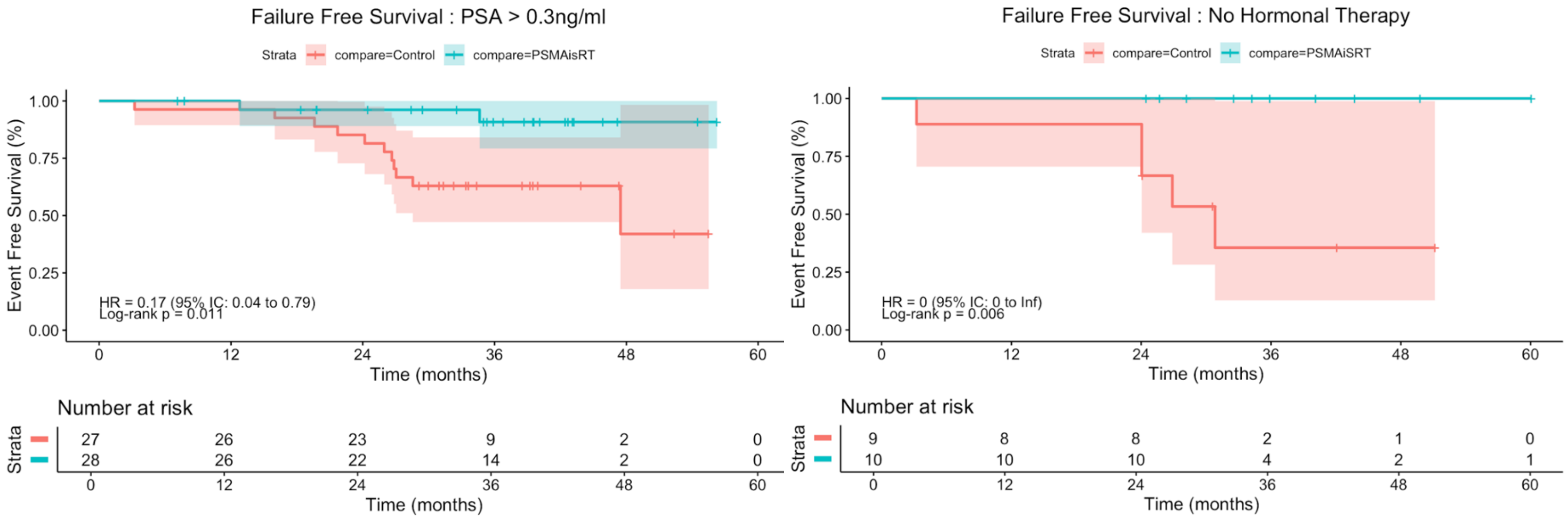

Kaplan meier curve : A) Failure Free Survival for patients with PSA ≥ 0.3 ng/mL before SRT, B) Failure Free Survival for patients treated without adjuvant HT therapy.

eTable 4. Frequency of grade 2+ and grade 3+ adverse events attributable to RT

		PSMAiSRT	Control	p
Acute (≤ 6 months)				
Overall	Grade 2+	27	31	0.58
	Grade 3+	1	1	1
Gastrointestinal	Grade 2+	14	10	0.49
	Grade 3+	0	0	1
Genitourinary	Grade 2+	10	14	0.49
	Grade 3+	1	0	1
Late (> 6 months)				
Overall	Grade 2+	32	29	0.72
	Grade 3+	5	2	0.44
Gastrointestinal	Grade 2+	9	6	0.58
	Grade 3+	1	0	1
Genitourinary	Grade 2+	16	13	0.67
	Grade 3+	2	1	1
Acute + Late				
Overall	Grade 2+	59	60	1
	Grade 3+	6	3	0.49
Gastrointestinal	Grade 2+	23	16	0.24
	Grade 3+	1	0	1
Genitourinary	Grade 2+	26	27	1
	Grade 3+	3	1	0.62

eTable 5. Patient-Based Cumulative Incidence of Grade 2+ and Grade 3+ Adverse Events for Event-Free Survival Analysis

		PSMAiSRT	Control	p
Acute (≤ 6 months)				
Overall	Grade 2+	17	23	0.34
	Grade 3+	2	1	1
Gastrointestinal	Grade 2+	11	10	1
Genitourinary	Grade 2+	7	12	0.32
Acute + Late				
Overall	Grade 2+	34	39	0.47
	Grade 3+	6	3	0.49
Gastrointestinal	Grade 2+	16	16	1
Genitourinary	Grade 2+	17	23	0.34

eFigure 2. Freedom From First CTCAEv.5 Event

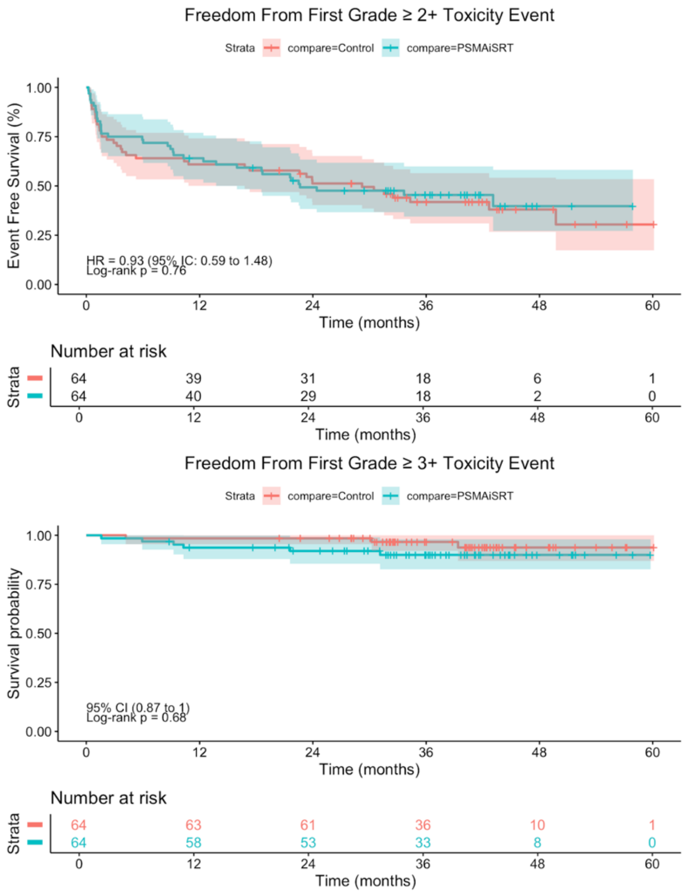

Kaplan Meier curves : A) Freedom from first grade 2+ toxicity event, B) Freeform from first grade 3+ toxicity.

eTable 6. MID events per EPIC-CP domain at different time points.

Domain / Time	MID event - PSMAiSRT<br>n=49	MID event - Control<br>n=38	p
Urinary incontinence			
12 months	10 events	5 events	0.35
24 months	17 events)	12 events	0.76
Urinary irritation / obstructive			
12 months	12 events	10 events	0.85
24 months	21 events	15 events	0.75
Bowel			
12 months	16 events	10 events	0.52
24 months	23 events	15 events	0.49
Sexual			
12 months	14 events	17 events	0.12
24 months	16 events	19 events	0.11
Hormonal			
12 months	19 events	19 events	0.30
24 months	23 events	22 events	0.31
