# Supplementary material for: Prostate-Specific Membrane Antigen PET-Guided Intensification of Salvage Radiotherapy After Radical Prostatectomy: A Phase 2 Randomized Clinical Trial
Source: JAMA Oncol. 2025 Oct 2;11(12):1431–8. doi: 10.1001/jamaoncol.2025.3746 (PMC12581501; doi:10.1001/jamaoncol.2025.3746)
Supplement: Supplement 3. — Data Sharing Statement [file jamaoncol-e253746-s003.pdf]

Data Sharing Statement

Data
Additional Information: Trial Registration : PSMA-PET/CT-Guided Intensification of Radiation Therapy for Prostate Cancer : cmRCT-NCT03525288
https://clinicaltrials.gov/study/NCT03525288?cond=NCT03525288&rank=1
Data available: Yes
Data types: Deidentified participant data
How to access data: Access will be granted to researchers who submit a methodologically sound proposal, for any type of analysis. Proposals should be directed to cynthia.menard@umontreal.ca
When available: With publication

Supporting Documents
Document types: None

Additional Information
Who can access the data: Researchers
Types of analyses: Combined Analyses
Mechanisms of data availability: after approval of a proposal
